# Impact of REM sleep deprivation and sleep recovery on circulatory neuroinﬂammatory markers

**DOI:** 10.5935/1984-0063.20190157

**Published:** 2021

**Authors:** Konakanchi Suresh, Vinutha Shankar, Dayanand CD

**Affiliations:** 1 Sri Devaraj Urs Medical College, Sri Devaraj Urs Academy of Higher Education and Research, Department of Physiology - Kolar - Karnataka - India.; 2 Sri Devaraj Urs Medical College, Sri Devaraj Urs Academy of Higher Education and Research, Department of Biochemistry - Kolar - Karnataka - India.

**Keywords:** REM Sleep Deprivation, Inﬂammation, Sleep Recovery, Neuron-Specific Enolase, Creatine Kinase-Brain Fraction, Lactate Dehydrogenase Brain Fraction

## Abstract

**Objectives:**

Sleep loss may contribute to neuroinﬂammation, which might increase neuroinﬂammatory markers such as neuron-speciﬁc enolase (NSE), creatine kinase-brain fraction (CK-BB), lactate dehydrogenase brain fraction (LDH-BB) in blood. Hence, we evaluated the effect of REM sleep deprivation and recovery on these markers.

**Material and Methods:**

Twenty-four adult male Sprague Dawley rats were grouped as control, environmental control, REM sleep deprivation, and 24 hour sleep recovery. The rats were sleep deprived for 72 hours and recovered for 24 hours. NSE, CK-BB, and LDH-BB levels in serum were measured using ELISA.

**Results:**

The serum NSE, CK-BB, and LDH-BB were signiﬁcantly higher in 72 hour sleep deprived group compared to control (*p*<0.01). After 24 hours of sleep recovery, the levels of NSE, CK-BB, and LDH-BB were comparable to control (*p*>0.05).

**Discussion:**

REM sleep deprivation increased serum NSE, CK-BB, and LDH-BB, which might be due to neural damage. However, 24 hours of sleep recovery restored these markers.

## INTRODUCTION

Sleep has physiological importance in maintaining the integrity of the central nervous system (CNS) for physical and mental performance. Consequences of sleep loss such as disturbances in metabolism, immune, neuroendocrine, autonomic, blood-brain barrier, and cognitive functions have been documented^1^. It has been illustrated that sleep loss is associated with increased neuronal metabolism, it results in the production of reactive oxygen species and decreased antioxidants levels, which intern leads to oxidative injury of nervous system^2^. Rapid eye movement (REM) sleep deprivation activates the sympathetic nervous system and hypothalamic-pituitary-adrenal axis, which results in the activation of immune functions of CNS^3^. The activation of the immune system increases pro-inflammatory (tumor necrosis factor α (TNF-α), interleukin 1β (IL-1β), and interleukin 6 (IL-6)) and reduced anti- inflammatory cytokines (interleukin 4 (IL-4), and interleukin 10 (IL-10)). The alteration in neural metabolism and pro- and anti-inflammatory cytokines might result in neural inflammation^4^. There is evidence that the neural damage caused by sleep deprivation can be compensated by rebound sleep (sleep recovery)^5^.

Several studies have shown that the neural damage leads to neuroinflammation, it will increase the levels of neuroinflammatory markers such as neuron specific enolase (NSE), creatine kinase brain fraction (CK-BB), glial fibrillary acidic protein and S100 calcium- binding protein B, etc., in blood circulation^6^. Measurement of these markers can give information on the extent of the neural damage^7^. NSE is an isoenzyme present in the cytosol of neuronal cells. Earlier studies suggested that the levels of NSE were elevated in traumatic brain injury, stroke, and related preclinical models. NSE is reported as an ideal circulatory neuroinflammatory marker, which can directly assess the damage of neurons^8^. In addition to NSE, other circulatory neuroinflammatory markers such as CK-BB and lactate dehydrogenase brain fraction (LDH-BB) were also elevated in cerebrospinal fluid and blood samples of a patient with traumatic brain injury (TBI) and meningitis^9^. CK-BB is an isoform of creatine kinase present in the brain; it is reliably used as a marker for measuring the neural damage due to neurological disorders. In line with NSE, evidence shows that CK-BB has a strong correlation with brain injury^7,9^. LDH is an enzyme that catalyzes the reversible conversion of lactate to pyruvate. LDH-1 is an isoform of LDH, which is majorly expressed in the brain. Studies have reported that the levels of LDH in serum were proportionate to the severity of the neural injury^10^.

Even though several studies highlight the increased levels of the neuroinflammatory markers in circulation during neural damage (meningitis, TBI, stroke, and neurodegenerative diseases), there is paucity on how potentially the acute sleep deprivation results in neuronal damage and its influence on circulatory neuroinflammatory markers. Measurement of these markers may possibly provide information on REM sleep deprivation induced neuroinflammation. Therefore, the present study was aimed to find the consequences of REM sleep deprivation on circulatory neuroinflammatory markers and the efficacy of sleep recovery.

## MATERIAL AND METHODS

The present study was approved by the Institutional Animal Ethics Committee (Ref. No.: IAEC/PHARMA/SDUMC/2017-18/05). All the experiments were performed at the Department of Physiology, Sri Devaraj Urs Medical College, Sri Devaraj Urs Academy of Higher Education and Research, Kolar, Karnataka, India. Twenty-four male Sprague Dawley rats aged between 12-14 weeks with a bodyweight of 150-200g were procured from Biogen Laboratory Animal Facility, Bangalore (CPCSEA Registration No.: 971/bc/06). The rats were maintained in polypropylene cages at 24±2 ºC by exploring the food and water in ad libitum.

### Experimental design

**Figure 1:**
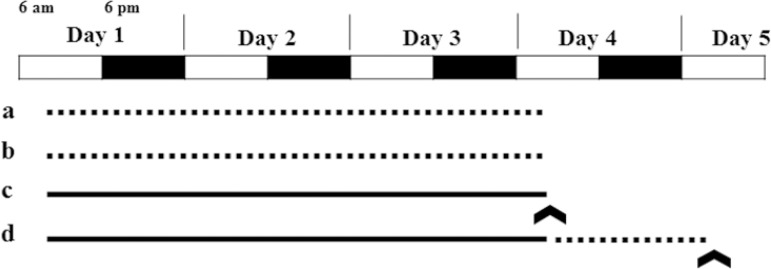
Overview of experimental design. a = control (rats placed in home cages), b = environmental control (rats exposure to stainless steel grid of MMPI for 72 hr), c = REM sleep deprivation (rats exposure to small platforms of MMPI for 72 hours, d = 24 hr sleep recovery group (rats placed in home cages for 24 hr after 72 hours exposure to small platforms of MMPI). Dotted lines (............) represents the normal sleep wake cycle, whereas, full line ( ________ ) represents the period of sleep deprivation, arrow ( ^ ) indicates the time of blood sample collection.

The rats were randomly grouped as control (n=6), environmental control (n=6), REM sleep deprivation (n=6) and 24 hour sleep recovery (n=6). A modified multiple platform instrument (MMPI) was used for the REM sleep deprivation^11^. The MMPI has 12 platforms (110x44x45cm), the diameter of each platform is 5.5cm and height is 6.0cm surrounded by water up to 1cm beneath the platform. Whenever the animal goes to sleep, it loses muscle tone and falls into the water and climbs back to the platform. In this method, animals were capable of moving inside the tank and jumping from one platform to another. The rats were habituated to MMPI for 3 days (daily one hour). Both REM sleep deprivation and sleep recovery group rats were sleep deprived by keeping in MMPI for 72 hours by supplying ad libitum of food, water and 12:12 hours (6 a.m. to 6 p.m.) of light (300 lux) and dark phases. The sleep recovery group rats were placed in their home cages for 24 hours for sleep recovery. Control rats were placed in their home cages without interruption of sleep. The environmental control group rats were kept for 72 hours in MMPI (stainless steel grid of 2.3mm pore size was placed 1cm above the water levels). In this setup, the animals can sleep on a stainless steel grid without falling into water^12^. The overall experimental design is shown in Figure 1.

### Collection of the blood sample

At the end of the intervention (Figure 1), the rats were anesthetized using a combination of ketamine and xylazine; 2ml of blood was collected through cardiac puncture. To avoid the influence of blood sampling time on the markers, blood samples from all the experimental groups were collected at a fixed time (between 10 to 11 a.m.). The blood samples were allowed to coagulate for 30 minutes. The serum samples were collected by centrifugation of blood at 3000rpm for 20 minutes at room temperature. Care was taken to avoid the hemolysis of the blood samples. The obtained serum was stored at -20 ºC until further analysis.

### Measurement of circulatory neuroinflammatory markers in serum

The levels of NSE and CK-BB in serum were measured using ELISA kits (Cat: BC-ER140914 and BC-ER141394, supplied by Biocodon technologies, USA) and LDH-BB was measured using ELISA kit (Cat: LT860101ETKKBA, Life Technologies, India) as per the manufacturer instructions. Briefly, the reagents, buffers, and samples were thawed to room temperature. 40µl of undiluted samples, 10 µl of respective antibodies, and 50 µl of streptavidin-horseradish peroxidase were added to the respective monoclonal antibody-coated microplate wells and incubated at 37 ºC for one hour. After incubation, the solution was discarded completely and wells were washed with a 1X washing buffer solution five times. To the wells, 50µl of chromogen reagent A and 50µl of chromogen reagent B were added and incubated for 10 minutes for developing yellow color. The reaction was stopped by adding 50µl of stop solution. The optical density of yellow color was measured at 450nm using ELISA reader (Rayto, RT-6100 microplate reader).

### Statistical analysis

Quantitative variables were represented as mean ± standard error of the mean. The mean circulatory neuroinflammatory markers obtained from different groups were compared by performing one-way ANOVA with Bonferroni corrections using SPSS software, version 20 (IBM, USA). The effect size was calculated using Cohen’s d formulae, we considered an effect size with an absolute value to be a smaller (>0.2 to <0.5), moderate (>0.5 to <0.8) and large (>0.8); *p*-value <0.05 was considered as statistically significant.

## RESULTS

### Variation in the serum levels of NSE in REM sleep deprived and recovered rats

**Figure 2:**
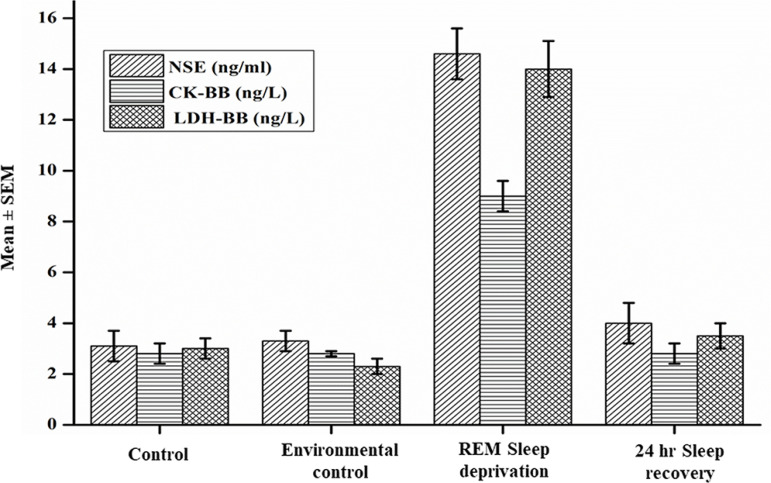
The serum levels of NSE, CK-BB, and LDH-BB among the control, environmental control, REM sleep deprivation, and 24 hour sleep recovery.

The mean ± SEM of NSE (ng/mL) obtained from control, environmental control, REM sleep deprivation and 24 hours sleep recovery were 3.1±0.6, 3.3±0.4, 14.6±1.0, and 4.0±0.8, respectively. Analysis of variance showed a significant increase in serum levels of the NSE in the REM sleep deprived group when compared to control and environmental control (*p*<0.01). The REM sleep deprived group subjected to 24 hours of sleep recovery showed a significant reduction (*p*<0.01) in the serum levels of NSE when compared to REM sleep deprived group. The serum levels of NSE in the sleep recovered group did not show a significant difference when compared to the control and environmental control groups (*p*>0.05). There was no significant difference in the serum levels of NSE between the control group and the environmental control group (*p*>0.05). Figure 2 shows the mean ± SEM of NSE in serum samples obtained from different groups.

### Variation in the serum levels of CK-BB in REM sleep deprived and recovered rats

The mean ± SEM of CK-BB (ng/L) obtained from the control, environmental control, REM sleep deprivation, and 24 hours sleep recovery were 2.8±0.4, 2.8±0.1, 9.0±0.6, and 2.6±0.6, respectively. Analysis of variance showed a significant increase in the serum levels of the CK-BB in the REM sleep deprived group when compared to control and environmental control (*p*<0.01). There was no significant difference in the serum levels of CK-BB between control and environmental control group (*p*>0.05). REM sleep deprived group subjected to 24 hours of sleep recovery showed a significant reduction (*p*<0.01) in the serum levels of CK-BB when compared to REM sleep deprived rats. The serum levels of CK-BB in sleep recovered group did not show a significant difference when compared to control and environmental control groups (*p*>0.05). Figure 2 shows the mean ± SEM of CK-BB in serum samples obtained from different groups.

### Variation in the serum levels of LDH-BB in REM sleep deprived and recovered rats

The mean ± SEM of LDH-BB (ng/L) obtained from the control, environmental control, REM sleep deprivation, and 24 hours sleep recovery were 3.0±0.4, 2.3±0.3, 14.0±1.1, and 3.5±0.5, respectively. There was no significant difference in the serum levels of LDH-BB between control and environmental control group (*p*>0.05). Analysis of variance showed a significant increase in the serum levels of the LDH-BB in the REM sleep deprived group when compared to control and environmental control groups (*p*<0.01). The REM sleep deprived group subjected to 24 hours of sleep recovery showed a significant reduction (*p*<0.01) in the levels of LDH-BB when compared to REM sleep deprived rats. The levels of LDH-BB in sleep recovered group did not show a significant difference when compared to the control and environmental control groups (*p*>0.05). Figure 2 shows the mean ± SEM of LDH-BB in serum samples obtained from different groups.

The effect size for serum levels of NSE, CK-BB, and LDH-BB obtained from control vs REM sleep deprivation, environmental control vs REM sleep deprivation, REM sleep deprivation vs 24 hour sleep recovery was greater than 0.9. Effect size of control vs environmental group, control vs 24 hour sleep recovery, environmental control vs 24 hour sleep recovery was between 0.2 and 0.5.

## DISCUSSION

In the present study, the rats were subjected to REM sleep deprivation for 72 hours, we observed an increased circulatory neuroinflammatory marker such as NSE, CK-BB, and LDH-BB in serum (Figure 2). The mean difference (control vs REM sleep deprivation and environmental control vs REM sleep deprivation) in these markers showed a large effect size, which indicates that the REM sleep deprivation significantly increased these markers. The present study results are similar to the study on humans subjected to sleep deprivation for one night, which showed a 20% increase of NSE in fasting blood samples^13^. Periasamy et al.^14^ reported that 72 hours of experimental sleep deprived mice showed a significant increase of LDH, creatinine kinase myocardial fraction, TNF-α, IL-6, and IL-1β in blood. Chennaou et al.^15^ reported that 24 hours of sleep deprivation increased the levels of TNF-α, IL-1β, and IL-6 in both hippocampus and peripheral blood. All these studies results demonstrate that sleep deprivation increases these markers in the circulation, which is an indication of neuroinflammation. There are evidences that sleep deprivation can generate excessive reactive oxygen species and decrease antioxidants resulting in mild neuroinflammation and neuronal damage^16,17^. In addition to neuroinflammation, He et al.^18^ reported that there was an increase in paracellular permeability of the blood-brain barrier after sleep deprivation. It is presumed that mild neuroinflammation with impairment of blood brain barrier functioning might be responsible for increasing the circulatory neuroinflammatory markers such as NSE, CK-BB, and LDH-BB in serum after 72 hours of REM sleep deprivation.

We have also observed that the 24 hours of sleep recovery reduced the circulatory neuroinflammatory markers (NSE, CK-BB, and LDH-BB) induced by REM sleep deprivation, which is comparable to controls (Figure 2). The mean difference (24 hour sleep recovery vs REM sleep deprivation) in these markers showed a large effect size, which indicates that the 24 hour sleep deprivation significantly decreased these markers. Mathangi et al.^19^ demonstrated that 24 hours of restorative sleep following 96 hours of REM sleep deprivation restores the oxidative stress markers levels to baseline. He et al.^18^ also reported that the permeability of the blood-brain barrier was attenuated within 24 hours of sleep recovery. In line with the published reports, our results demonstrated that REM sleep deprivation restored the circulatory levels of neuroinflammatory markers.

The present study results can be interpreted as consequences of neuroinflammation. Though, one should not rule out the role of systemic inflammation (cardiac muscle, skeletal muscle, and leucocytes) in the increase of circulatory neuroinflammatory markers in serum. However, we followed the standard methods to sleep deprive the animals and the observed increased levels of these markers are similar to the available literature (Benedict et al., 2014^13^ and Chennaoui et al., 2015^15^), so we presume that the variation of these markers is due to the neuro inflammation. Simultaneous measurement of these markers in blood and CSF can rule out the systemic changes, which will give us a better understanding of the REM sleep deprivation induced neural damage.

## CONCLUSION

The results showed that the serum neuroinflammatory markers are elevated after REM sleep deprivation, which might be indirectly related to neuronal damage and the change in levels of these markers is reversed on sleep recovery.
